# Gases in Sepsis: Novel Mediators and Therapeutic Targets

**DOI:** 10.3390/ijms23073669

**Published:** 2022-03-27

**Authors:** Zhixing Zhu, Stephen Chambers, Yiming Zeng, Madhav Bhatia

**Affiliations:** 1 Department of Pathology and Biomedical Science, University of Otago, Christchurch 8140, New Zealand; zhixing.zhu@postgrad.otago.ac.nz (Z.Z.); steve.chambers@otago.ac.nz (S.C.); 2 Department of Internal Medicine (Pulmonary and Critical Care Medicine), The Second Clinical Medical School of Fujian Medical University, Quanzhou 362002, China; zeng_yi_ming@126.com

**Keywords:** sepsis, pathophysiology, gaseous mediators, NO, CO, H_2_S, therapeutic targets

## Abstract

Sepsis, a potentially lethal condition resulting from failure to control the initial infection, is associated with a dysregulated host defense response to pathogens and their toxins. Sepsis remains a leading cause of morbidity, mortality and disability worldwide. The pathophysiology of sepsis is very complicated and is not yet fully understood. Worse still, the development of effective therapeutic agents is still an unmet need and a great challenge. Gases, including nitric oxide (NO), carbon monoxide (CO) and hydrogen sulfide (H_2_S), are small-molecule biological mediators that are endogenously produced, mainly by enzyme-catalyzed reactions. Accumulating evidence suggests that these gaseous mediators are widely involved in the pathophysiology of sepsis. Many sepsis-associated alterations, such as the elimination of invasive pathogens, the resolution of disorganized inflammation and the preservation of the function of multiple organs and systems, are shaped by them. Increasing attention has been paid to developing therapeutic approaches targeting these molecules for sepsis/septic shock, taking advantage of the multiple actions played by NO, CO and H_2_S. Several preliminary studies have identified promising therapeutic strategies for gaseous-mediator-based treatments for sepsis. In this review article, we summarize the state-of-the-art knowledge on the pathophysiology of sepsis; the metabolism and physiological function of NO, CO and H_2_S; the crosstalk among these gaseous mediators; and their crucial effects on the development and progression of sepsis. In addition, we also briefly discuss the prospect of developing therapeutic interventions targeting these gaseous mediators for sepsis.

## 1. Introduction

Sepsis is a life-threatening organ dysfunction that arises as a consequence of the host response failure to control invading pathogens, including bacteria, viruses, fungi and protozoa, as well as their toxins and subsequent dysregulation of the immune response. Septic shock refers to a subgroup of sepsis with a higher risk of mortality due to severe circulatory failure, in addition to cellular and metabolic abnormalities [1,2]. Sepsis/septic shock develops in approximately 30 million people every year, and the reported incidence of sepsis/septic shock keeps rising, making sepsis/septic shock a major health crisis worldwide [3,4]. Notably, sepsis/septic shock is not only responsible for about 20% of deaths worldwide, but it also takes a heavy toll on the quality of life of survivors. Patients who survive sepsis/septic shock usually suffer from post-sepsis sequelae, including neurocognitive impairment and psychological deficits [2]. As a result, sepsis/septic shock has been identified as a leading cause of morbidity, mortality and disability [4,5,6]. As such, sepsis/septic shock has become a global health priority since 2017 [5]. However, the development of effective therapeutic agents for sepsis/septic shock is still an unmet need and a great medical and scientific challenge. In order to address this health burden, gaining an in-depth understanding of the mediators involved in sepsis/septic shock pathogenesis, together with the biomarkers and therapeutic targets of sepsis/septic shock, is urgently needed.

Gaseous mediators refer to several gaseous signaling messengers that are endogenously and enzymatically produced [7,8]. Most of their functions do not occur when they are in a gaseous state, although they are collectively termed gaseous mediators [9,10,11,12,13]. In recent years, gases, such as sulfur dioxide, hydrogen cyanide and methane, have emerged as potential novel gaseous mediators [8,14,15,16]; however, to date, only three small, chemically reactive and freely permeable molecules comprise the novel signaling gaseous mediator family, which includes nitric oxide (NO), carbon monoxide (CO) and hydrogen sulfide (H_2_S) [8]. Importantly, NO, CO and H_2_S harbor several features in common. Firstly, these gaseous mediators are widely distributed in various tissues and organs. In addition, the biological half-life of each gas is short, with a range from seconds to minutes. Moreover, although they are hazardous at moderate to high concentrations, they play multiple, indispensable roles in a wide range of critical cellular functions and biological processes in low amounts. Consequently, any disturbance with respect to the homeostasis of these gaseous mediators, including their metabolism and bioavailability, can profoundly affect various physiological and pathological functions in the body, thereby leading to the onset and progression of various pathological conditions [17,18]. Recently, several studies have appeared that indicate NO, CO and H_2_S may be involved in the development of different stages of sepsis/septic shock [19,20,21]. However, the ways in which these gaseous signaling molecules affect the pathophysiology of sepsis/septic shock are not yet fully clear.

A comprehensive review focusing on summarizing and comparing the roles of these three gaseous messengers in sepsis/septic together would contribute to bridging the gaps in our knowledge. In this review, we aim to summarize the state-of-the-art knowledge on the pathophysiology of sepsis/septic shock and provide an update of the biometabolism, bioavailability and biofunction of NO, CO and H_2_S, together with the potential interaction among these gaseous mediators. Moreover, we attempt to emphasize the present-day evidence that points to the potential effects of these gaseous mediators on the development and progression of sepsis/septic shock. Additionally, we seek to briefly discuss the prospect of developing therapeutic interventions for sepsis by targeting these gaseous signaling molecules.

## 2. Pathophysiology of Sepsis

Sepsis affects nearly every organ system in living organisms; however, the lungs, liver, kidneys, heart, central nervous system and hematologic system are found to be most vulnerable and are frequently affected in sepsis [22]. Of note, as a well-characterized hallmark of sepsis, multiple organ dysfunction syndrome serves as a major determinant of the course and outcome of sepsis [23,24]. However, the underlying mechanism by which multiple organ dysfunction syndrome occurs in sepsis is far from fully understood [25].

**Figure 1:**
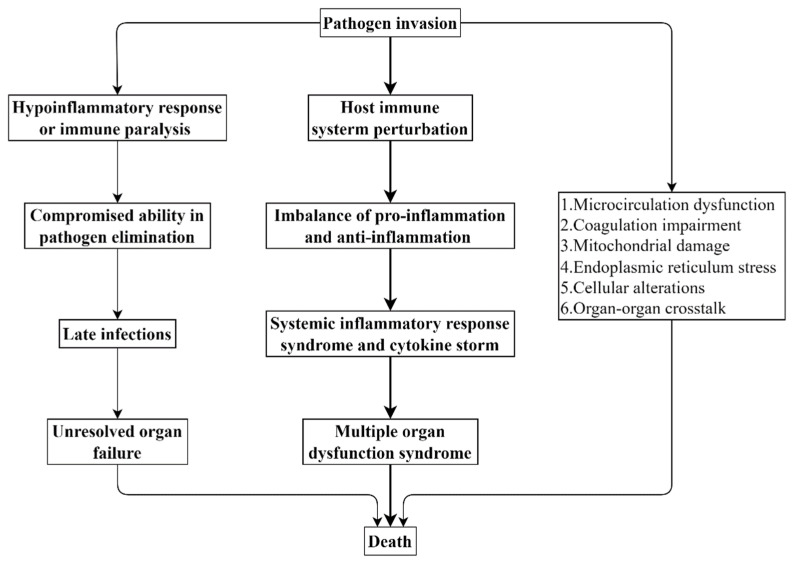
Pathophysiology of sepsis.

Many researchers have centered their focus on the involvement of a dysregulated host defense response in sepsis. A normal host defense response to infection can localize and control pathogen invasion and simultaneously initiate the repair process at the site of inflammation. However, the host immune system is profoundly perturbed in sepsis; thus, the host defense response in sepsis appears to be inappropriate and detrimental [26,27]. Inflammation plays a critical role in many other pathological conditions, such as cancer [28]. Specifically, the devastating infection caused by these invasive pathogens can lead to a multifaceted imbalance of proinflammatory response and anti-inflammatory response in the body [29,30]. To make matters worse, this destructive and uncontrolled host defense response can further result in systemic inflammatory response syndrome and cytokine storm, thereby ultimately progressing to multiple organ failure or even death [29,31]. As a result, sepsis has been treated as an exaggerated inflammation-led clinical trajectory from local microorganism infection to multiple organ dysfunction or death [22]. On the other hand, upon infection, the hypoinflammatory response/immune paralysis termed “endotoxin tolerance” is triggered simultaneously with the activation of proinflammatory response [32,33]. Due to the concomitant paralyzed immune system, the host’s ability to eradicate the invading microbes is severely compromised, which opens the way for late infections to develop and leads to unresolved organ failure. Hence, a prolonged and disorganized host hypoinflammatory response/immune paralysis is also detrimental and contributes to the increased risk of death in sepsis [34,35]. On top of the extensive involvement of an overactive host defense response, the importance of microcirculation dysfunction, coagulation impairment, mitochondrial damage, endoplasmic reticulum stress, cellular alterations and organ–organ crosstalk is also becoming increasingly evident and recognized [24,25] (Figure 1).

## 3. Gaseous Mediators

NO, CO and H_2_S are known for their toxic activity, but they are believed to have contributed to the origin of life and the appearance of eukaryotic animals, which should not be ignored [13]. More importantly, NO, CO and H_2_S are known to play crucial roles in diverse physiological and pathological conditions. To that end, increasing attention has been paid to these gaseous signaling mediators.

### 3.1. Nitric Oxide (NO)

NO, a colorless and toxic gas with a slight odor, was the first identified gaseous mediator. The endogenous synthesis of NO in eukaryotic cells is predominantly catalyzed by nitric oxide synthase (NOS) isozymes [36]. To date, three distinctive NOS isoforms comprise this enzyme family in mammals, which are neuronal NOS (nNOS), endothelial NOS (eNOS) and inducible NOS (iNOS) [37]. While the isoforms of nNOS and eNOS are calcium dependent and are constitutively expressed in cells, the iNOS isoform is calcium independent, and its expression is upregulated in response to many proinflammatory stimuli, including cytokines and lipopolysaccharide (LPS) [37,38]. More recently, the mitochondrial NOS (mtNOS) isozyme has been identified. This is also calcium dependent and constitutively expressed. Even though it is still somewhat controversial, this enzyme is emerging as the fourth member of the NOS family, since it is capable of catalyzing the synthesis of NO [39]. It is noteworthy that all these enzymes catalyze the same stepwise biological process. With the catalysis of these enzymes, L-arginine is hydroxylated and eventually converts to L-citrulline in the presence of molecular oxygen, nicotinamide adenine dinucleotide phosphate (NADP) and several cofactors, such as tetrahydrobiopterin (BH4). From this two-step enzymatic reaction, NO is liberated [40,41]. NO can be further oxidized to stable end products, namely nitrite and nitrate. Recently, it turned out that nitrate and nitrite are not only the oxidation products of NO but also the major biological reserves of NO [42]. On top of the oxidation of L-arginine, NO can be generated by the reduction of nitrite and nitrate. This noncanonical pathway is thought to be the compensatory mechanism for NO synthesis under hypoxic and acidic circumstances [42,43]. As a gaseous free radical, NO is highly reactive [44].

NO is best known as an endothelium-derived relaxing factor due to its role in mediating the dilation of blood vessels [8,45,46]. NO is also responsible for the regulation of vascular tone, platelet–vessel interactions, blood flow, angiogenesis and heart function [45,46,47,48]. Therefore, NO is very important, as the cardiovascular system is involved in various pathological conditions, including cancer [49]. Apart from this, as a versatile cellular signaling messenger, NO exerts a wide range of functions in many other organ systems, such as the nervous system, endocrine system, respiratory system, immune system and digestive system. Specifically, NO plays a significant role in the regulation of nerve development and neurotransmission, insulin secretion, airway tone, immune response reaction, wound healing and intestinal peristalsis [36,50]. In addition, it is becoming apparent that NO also functions as an endogenous regulator in epigenetics, including DNA methylation, DNA demethylation and histone post-translational modifications [51]. Moreover, NO has been shown to counteract oxidative stress, inhibit cell death and facilitate pathogen scavenging [44]. Dysregulated metabolism of NO is central to the pathogenesis of many diseases, including, but not limited to: sepsis, cancers, hypertension, stroke, inflammation, diabetes and retinopathy [19,36,52,53].

### 3.2. Carbon Monoxide (CO)

CO, a colorless, poisonous and odorless gas, was recognized as a gaseous mediator following NO. The majority of endogenous CO production is catalyzed by heme oxygenase (HO) isozymes [54,55]. Similar to NOS isozymes, this enzyme family also encompasses three different isoforms, namely HO-1, HO-2 and HO-3 [55]. HO-1 and HO-2 are two common and functionally active isoforms of HO. Specifically, HO-1, also known as heat shock protein 32, is inducible and highly dynamic; however, HO-2 is constitutively expressed, and its expression is much less regulated [44,56]. Controversially, a new isoform of HO was discovered in rat tissues and was referred to as HO-3 [56]. Despite HO-3 having significant homology to HO-2 in the amino acid sequence (as high as 90%), its enzyme activity is much inferior to that of HO-2 [55,56]. CO is produced during the degradation of heme. Specifically, HO-catalyzed CO synthesis starts with the catabolism of heme. In this process, heme is firstly oxidized to α-metahydroxyheme in the presence of oxygen and NADP; afterward, α-metahydroxyheme further reacts with oxygen, from which CO is produced [57]. This HO-catalyzed heme catabolism is biologically crucial, as this reaction is responsible for around 85% of CO production in living organisms. In addition to HO, many other enzymes, including cytochrome P450 reductase, human acireductone dioxygenase and tyrosinase, also contribute to the endogenous production of CO. Compared with heme-independent CO synthesis, heme-independent CO production, namely the oxidation of phenols, the peroxidation of lipids and the photo-oxidation of organic compounds, is minor; however, these reactions are also contributory [54,55]. Although CO can be further oxidized to CO_2_, the oxygenation of CO in mammals under normal conditions has not been observed. Unlike NO, the predominant catabolism of endogenous CO is either by exhalation (approximately 80%) or by binding to hemoglobin and other heme proteins.

CO is bioactive and plays a pivotal role in a variety of biological systems [58,59]. In the cardiovascular system, although it is not as potent as NO, CO also serves as a vasodilator in the body because of its role in inducing the relaxation of vascular smooth muscle. In addition, CO has been found to promote angiogenesis and inhibit the aggregation and adhesion of platelets and the activation of monocytes [44,60]. In the respiratory system, CO acts as a crucial bronchodilator, as it is essential for reversing methacholine-induced bronchoconstriction and relaxing tracheal smooth muscle [61]. In the digestive system, CO has been found to be gastroprotective due to its involvement in maintaining gastric mucosal integrity and promoting gastric ulcer healing [62]. CO also acts as a neurotransmitter due to its versatile roles in regulating the functions of the nervous system, such as neurodevelopment, long-term memory and sensory discharge [44]. CO is also an endogenous modulator of inflammation, cell death, oxidative stress and immune responses [10,63]. Alternations in CO metabolism have been observed in various pathological disturbances, such as sepsis, lung injury, anemias and liver cirrhosis [64,65,66].

### 3.3. Hydrogen Sulfide (H_2_S)

H_2_S, a colorless, flammable and notorious gas with a pungent odor of rotten eggs, has emerged as the third gaseous signaling mediator. The endogenous generation of H_2_S in mammalians mainly arises from the desulfydration of L-cysteine or homocysteine. To date, three enzymes with ascribed roles in this conventional source of H_2_S biosynthesis have been recognized. These are cystathionine β-synthase (CBS), cystathionine γ-lyase (CSE) and 3-mercaptopyruvate sulfurtransferase (3-MST) [67,68,69]. Among these enzymes, CBS and CSE, two pyridoxal 50-phosphate (vitamin B6)-dependent enzymes, catalyze transsulfuration reactions, whereas 3-MST is responsible for L-cysteine catabolism [70]. Specifically, CBS, the first enzyme in the transsulfuration pathway, generates H_2_S, mainly by the condensation reaction of L-cysteine and homocysteine, while CSE, the second enzyme in the pathway, produces H_2_S, predominantly by the α, β-elimination of L-cysteine [67,71]. While CBS is believed to be the main source of H_2_S biosynthesis in the central nervous system, CSE is thought to be the major contributor to endogenous production of H_2_S in the peripheral tissues [67,72]. As the third enzyme involved in H_2_S biosynthesis, 3-MST, along with cysteine aminotransferase (CAT), also contributes to the endogenous production of H_2_S [72]. Particularly, 3-MST-catalyzed H_2_S generation requires the presence of reducing substrates, and most of the yielding H_2_S is bound in the form of sulfate sulfur. While CBS and CSE are primarily cytosolic enzymes, 3-MST is mainly localized in the mitochondrion and with some in the cytoplasm. Consequently, CBS- or CSE-catalyzed H_2_S biosynthesis mainly occurs in the cytoplasm, whereas 3-MST-mediated H_2_S generation takes place in both places [70]. More recently, the production of H_2_S by 3-MST and the D-amino acid oxidase (DAO) pathway has been identified [73]. Apart from these enzyme-catalyzed reactions, it has become increasingly apparent that many other sources, including the natural liberation of H_2_S from persulfides and polysulfide species, also contribute to H_2_S biosynthesis [74]. The predominant pathways by which H_2_S is metabolized in vivo include oxidation to thiosulfate and sulfate in the mitochondrion and methylation to methanethiol in the cytoplasm. In addition, H_2_S can be eliminated by methemoglobin or metallo- or disulfide-containing molecules. Moreover, thiosulfate and sulfate excretion by urinating also leads to the clearance of H_2_S [69,75]. Unlike its two counterparts, NO and CO, which employ soluble guanylyl cyclase to transduce their signals, it is yet not clear whether H_2_S also has its second messenger. Nonetheless, as a gaseous free radical similar to NO, H_2_S is also biologically reactive [76].

H_2_S has been found to be a versatile modulator of various organs and systems [77]. In the context of a pivotal role of an endothelium-derived relaxing factor for NO, H_2_S is regarded as an endothelium-derived hyperpolarizing factor [46,78]. However, H_2_S also possesses a potent vasoconstrictive effect [79]. More significantly, it is reported that endogenous H_2_S can regulate the bioavailability of NO in the cardiovascular system [80]. Apart from the regulatory effect on vascular tone, H_2_S also participates in regulating the proliferation and death of vascular smooth muscle cells, inhibiting oxidative stress suppression inflammation inhibition and modulating vascular permeability and angiogenesis [81]. In the respiratory system, H_2_S profoundly affects various respiratory functions, such as regulating the respiratory rhythm and maintaining the development and homeostasis of the pulmonary vessel [82,83]. In the immune system, H_2_S regulates the viability and functions of various immune cells, including neutrophils, macrophages, dendritic cells, T lymphocytes and B lymphocytes; thus, it greatly shapes the landscape of innate and adaptive immunity [84]. In the central nervous system, H_2_S serves as an antioxidant and antineuroinflammatory mediator; thus, H_2_S plays a significant role in neuroprotection. Moreover, H_2_S is closely associated with neurotransmission [85]. H_2_S also affects the functions of many other systems, such as the reproductive system, digestive system and endocrine system [77]. Notably, similar to NO, H_2_S is discovered to be an important endogenous epigenetic modulator [51]. Aberrant H_2_S metabolism occurs in many pathological states, such as sepsis, inflammation, coronavirus disease 2019, hypertension, atherosclerosis, obstructive respiratory disease, lung injury, macrophage activation, retinal diseases and neurodegenerative disease [77,81,86,87,88,89].

### 3.4. Interplay among NO, CO and H_2_S

Accumulating evidence has pointed to the crosstalk among NO, CO and H_2_S [86,90]. To date, four interaction mechanisms have been identified, which are competition/synergy for heme in heme-containing proteins, modulation of the generation of other gases and competition for post-translational modification sites in proteins and formation of hybrid molecules [91,92,93]. Among these four mechanisms, the regulation of the biosynthesis of other gaseous mediators has been widely explored. Firstly, NO has been shown to upregulate the endogenous production of CO by increasing the level of HO-1, whereas NO can suppress the activity of HO-2 [94,95]. The regulatory effects of NO on H_2_S generation are also complex. While NO has been shown to inhibit the activities of CBS and CSE, it can also promote CSE-mediated H_2_S production [96,97,98]. Secondly, CO has been shown to reduce the production of NO and H_2_S by suppressing the activities of iNOS and CBS [99]. Thirdly, H_2_S also greatly affects the biosynthesis of NO and CO. H_2_S can promote or stabilize eNOS activity; however, it can also inhibit eNOS function [97,100,101]. Additionally, in the presence of NO, the activities of nNOS and iNOS were suppressed by H_2_S [102]. Moreover, H_2_S can inhibit iNOS activity by promoting HO-1 activity [103]. However, NO production can be enhanced by H_2_S as it can promote iNOS expression [104]. Importantly, the crosstalk among NO, CO and H_2_S may differ in different organs or under different conditions, leading to the complicated or even opposite regulatory effects of one gaseous mediator on the biosynthesis of others.

Table 1 briefly summarizes the characteristics of NO, CO and H_2_S.

**Figure 2:**
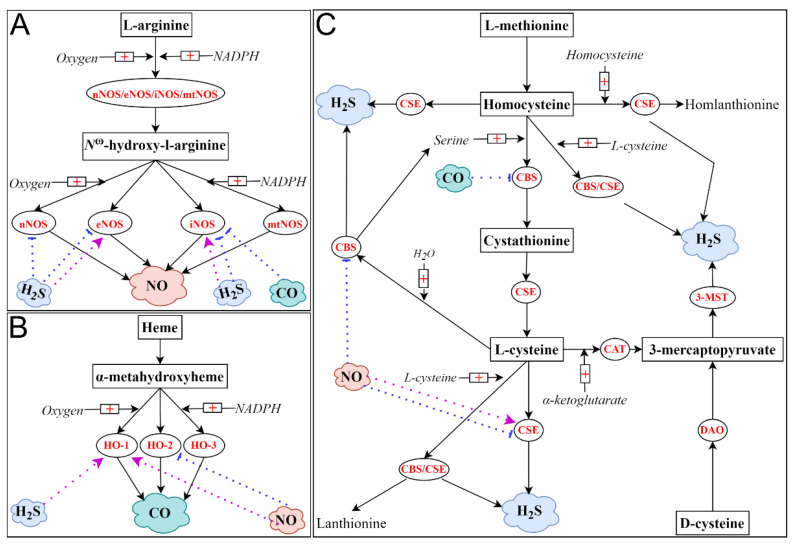
Enzyme-derived endogenous production of NO (**A**), CO (**B**) and H_2_S (**C**). Endogenous NO is produced from a two-step biological process catalyzed by NOS. L-arginine is hydroxylated to *N*^ω^-hydroxy-l-arginine; the latter is further oxidized to L-citrulline and NO. Endogenous CO is generated from the degradation of heme. Firstly, heme is oxidized to α-metahydroxyheme in the presence of oxygen and NADPH. Secondly, α-metahydroxyheme reacts with oxygen, resulting in the biosynthesis of CO. NADHP is also required for this reaction. Relatively, the biosynthesis of H_2_S is more complex as H_2_S can be generated from four enzymatic pathways. Briefly, H_2_S is naturally produced from the desulfydration of L-cysteine or homocysteine catalyzed by CBS, CSE and 3-MST/CAT. Specifically, CBS and CSE are involved in transsulfuration reactions, whereas 3-MST is responsible for L-cysteine catabolism. Recently, the 3-MST/DAO pathway has gained acceptance as the fourth pathway for H_2_S biosynthesis, using D-cysteine as the substrate. The biosynthesis of every gaseous mediator might be affected by the remaining two gases. Purple arrow: activation; blue arrow: suppression.

Figure 2 briefly summarizes the ways in which NO, CO and H_2_S are enzymatically produced and that in which the biosynthesis of one gaseous mediator is affected by the remaining two.

## 4. Gaseous Mediators in Sepsis/Septic Shock

As three significant endogenous regulators that play indispensable roles in maintaining the homeostasis of organ systems in living organisms [18], NO, CO and H_2_S have been shown to play vital roles in the intricate pathophysiology of sepsis/septic shock.

### 4.1. NO in Sepsis/Septic Shock

Overproduction of NO throughout the organism resulting from the excessive activation of iNOS is one hallmark of sepsis. NO has emerged as a significant modulator in sepsis, as it has been shown to extensively impact the pathophysiology and outcome of sepsis [105,106]. The level of NO was correlated with the increased severity of sepsis- and endotoxemia-associated systemic inflammation and organ injury, while the inhibition of NO production mitigated these alterations [107,108,109]. Specifically, in rats with cecal ligation and puncture (CLP)-induced sepsis or LPS-induced endotoxemia, the level of iNOS in the diaphragm was upregulated. The elevated iNOS and attendant increased production of NO were involved in endotoxemia- and sepsis-induced diaphragm injury, as alterations that occurred in the diaphragm (sarcolemmal injury and myofiber damage) were obviously mitigated by the administration of a nonselective NOS inhibitor named L-NMMA [107]. In addition, it was reported that the level of NO was elevated in mice with *Escherichia coli* infection-induced sepsis, and the increased NO production was associated with sepsis-associated alterations, such as dysregulated systemic inflammation, as indicated by elevated proinflammatory mediators, oxidative stress (increased malondialdehyde) and organ dysfunction (liver failure and kidney failure). However, the pretreatment of L-NAME, a nonselective inhibitor of NOS, significantly reduced sepsis-induced overproduction of NO and consequently mitigated sepsis-associated abnormalities [108]. Moreover, as shown by Luo et al., once the activity of the toll-like receptor 4 (TLR4)/myeloid differentiation primary response 88 (MyD88)/nuclear factor kappa-light-chain-enhancer of activated B cells (NF-κB) pathway was inhibited, iNOS became inactive, leading to an enhancement of vascular responsiveness and an increase in the survival of mice with CLP-induced sepsis (mean survival time increased from 1.7 days to 4.5 days) [109]. Similarly, compared with patients without sepsis, the concentration of serum NO in sepsis patients was significantly upregulated [110]. More importantly, monitoring of the level of NO in serum may contribute to precise of evaluation the severity of sepsis [110]. Among the organs and systems affected by elevated NO in sepsis and endotoxemia, the cardiovascular system has attracted the most attention. While a physiological level of NO is essential to the maintenance of the cardiovascular system, sepsis-associated hemodynamic instability, including vasorelaxation, hypotension and shock, has been attributed to an aberrant NO-induced macrovascular compromise, myocardial dysfunction, vascular hyporesponsiveness, direct cellular toxicity and bioenergetic failure [47]. Furthermore, when the amount of endogenous NO reaches a certain threshold, the production of NO is inhibited, since the high concentration of NO itself can suppress the activity of NOS [37]. Moreover, compared with iNOS, nNOS and eNOS are more sensitive to the autoinhibitory effect of NO. Therefore, once the biosynthesis of NO catalyzed by iNOS is activated in response to the proinflammatory stimuli in sepsis, the essential basal effects of eNOS may be insufficient to support the function of the fragile cardiovascular system in sepsis [46,105]. Thus, the aberrant production of NO catalyzed by active iNOS is thought to be a primary cause of hemodynamic instability in sepsis [47,111]. Worse still, these NO-induced hemodynamic alterations can further result in hypoxia in multiple organ systems, leading to progressive organ dysfunction [112]. In addition, many studies have shown that there is extensive involvement of NO in sepsis- and endotoxemia-induced abnormalities and derangements in the respiratory system, renal system, immune system, central nervous system and digestive system and identified the underlying mechanisms by which these organs and systems are disturbed by dysregulated NO in sepsis and endotoxemia [105].

Not surprisingly, several studies have also concluded that the increased generation of NO may have potential benefits in sepsis, since NO has been shown to facilitate bacterial destruction [112]. For example, in a controlled trial of inhibition of nNOS either by pharmacological blockage or gene deletion, there was an increase in mortality (hazard ratio of death was 1.71) and blood bacterial counts (1.4-fold greater) in mice with sepsis induced by CLP. This was accompanied by an upregulation of proinflammatory mediators, including tumor necrosis factor (TNF)-α and interleukin (IL)-6, and peritoneal lavage cell counts were increased. These results indicate that the nNOS/NO pathway improves survival from sepsis plays an important role in modulating the inflammatory response [113]. In another study, sepsis was induced in wild-type mice and genetically deficient iNOS-knockout mice by infection with *Salmonella typhimurium* (a Gram-negative pathogen). The deletion of the iNOS gene attenuated sepsis-induced systemic inflammation, as evidenced by lower levels of proinflammatory mediators and neutrophil accumulation in the peritoneal cavity. In addition, the deficiency of the iNOS gene also increased the bacterial load, decreased the thymic atrophy, aggregated the hepatic and cardiovascular dysfunction and increased the risk of mortality of mice. By contrast, the pretreatment of iNOS-deficient mice with DETA-NO (a NO donor) significantly attenuated these sepsis-associated abnormalities. These findings revealed the protective roles played by the iNOS/NO pathway in sepsis [114].

### 4.2. CO in Sepsis/Septic Shock

Increased generation of CO is also commonly observed in sepsis and endotoxemia, and many studies have implicated the beneficial effects of CO in sepsis [115]. Kyokane et al. reported that endogenous production of CO catalyzed by HO-1 in the liver was upregulated in rats with LPS-induced endotoxemia and concluded that the increased production of CO played an important role in protecting the liver from dysfunction [116]. Similarly, HO-1-derived CO resulted in an enhancement of phagocytosis and host defense response directed at microorganism invasion, leading to an enhancement of pathogen clearance without suppressing the host inflammatory response in CLP-induced sepsis in mice. In addition, the administration of the tricarbonyldichlororuthenium (II) dimer, a CO-releasing molecule (CORM), significantly improved the survival probability of mice in sepsis [117]. The protective role of CO has been confirmed in CLP-induced sepsis, as well as LPS-induced endotoxemia, both in vivo and in vitro [118]. Specifically, in vivo, the administration of HO-1 inducers or CORM-2 suppressed the activity of high-mobility group box 1, improving the survival of sepsis. In addition, sepsis-associated systemic inflammation was alleviated by the treatment of HO-1 inducers and CORM-2, as indicated by a drop of proinflammatory cytokines, including TNF-α, IL-1β and interferon-β. In vitro, the induction of LPS-induced endotoxemia activated the activity of high-mobility group box 1, thereby promoting the proinflammatory response in macrophages. However, the pretreatment of HO-1 inducers or CORM-2, as well as the transfection of HO-1, greatly reversed these alterations [118]. Since several CORMs have been developed [119], many researchers have used different CORMs in their research, and the protective roles of the HO-1/CO pathway in sepsis and endotoxemia have been firmly established. For example, several studies showed that the CO released from CORM-2 or CORM-3 was capable of suppressing the activation of inflammasome related to pyroptosis; as a result, the function of multiple organs and systems (cardiac fibroblasts, intestine and kidney) were preserved, and the elimination of pathogens was promoted in rodents with CLP-induced sepsis [120,121,122,123]. Apart from the effects on dampening proinflammatory response and inhibiting pyroptosis, many other mechanisms, such as supporting the energetic metabolism of mitochondrion coupled with activating the biogenesis of mitochondria, reducing the levels of cardiac contractile proteins, inhibiting the activation of NF-κB, downregulating the expression of the TLR4/myeloid differentiation factor-2 (MD2) complex on myeloid cells, suppressing the overactivation of platelets and enhancing autophagy, also contribute to the protective actions of CO in sepsis and endotoxemia [124,125,126,127,128,129]. Of note, the nuclear factor-erythroid factor 2-related factor 2 (Nrf2) has been shown to be essential to the anti-inflammatory roles of CO released from CORM-2 in LPS-induced endotoxemia, as the deletion of Nrf2 significantly abolished the beneficial effects of CO [130]. Interestingly, exposure of mesenchymal stromal cells to CO enhanced the therapeutic response in mice with CLP-induced sepsis [131]. This study showed that CO exposure greatly improved the treatment efficacy of mesenchymal stromal cells, as these cells have been shown to enhance pathogen elimination, promote inflammation resolution and alleviate organ injury in septic mice. Consistently, increased biosynthesis of CO has also been observed in septic patients; more importantly, survivors had higher levels of CO than nonsurvivors, indicating the beneficial effect of increased CO production in sepsis [64,132].

Only a few investigations have demonstrated that the upregulation of endogenous CO is detrimental to sepsis. For instance, Iwasashi and coworkers found that the active HO-1/CO pathway was associated with liver dysfunction in rats subjected to CLP-induced sepsis. It was reported that HO-1-induced excessive generation of CO led to an immoderate dilation of liver sinusoidal and attendant liver failure, whereas the administration of HO inhibitors (Sn-PP and Zn-PP) significantly alleviated sepsis-induced liver injury, as evidenced by lower plasma aspartate aminotransferase and lower liver cyclic guanosine monophosphate, as well as promoted the survival of rats (61.5% and 66.7% vs. 26.7%) [133].

### 4.3. H_2_S in Sepsis/Septic Shock

The biosynthesis of H_2_S is significantly upregulated in sepsis. Accumulating evidence has shown proinflammatory effects of H_2_S on sepsis and endotoxemia. Induction of septic shock and endotoxic shock has been reported to greatly increase the arterial level of H_2_S in rats, and the elevated H_2_S has a negative correlation with the hemodynamic parameters, including the heart rate, the mean arterial pressure and the +d*P*/dt max in rats [134]. This investigation aroused great attention in exploring the roles of H_2_S in sepsis and the mechanisms of action. As showed in a landmark study that detailed the significant role of endogenous H_2_S in CLP-induced sepsis, the expression (both mRNA and protein) of CSE and the level of endogenous H_2_S were greatly upregulated after the induction of sepsis [135]. The administration of DL-propargylglycine (PAG, 50 mg/kg, intraperitoneal injection), an irreversible inhibitor of CSE, significantly attenuated sepsis-induced neutrophil accumulation, as indicated by tissue myeloperoxidase activity and histological alterations in the liver and lungs, whereas the treatment of sodium hydrosulfide (NaHS, 10 mg/kg, intraperitoneal injection), a fast-releasing H_2_S donor, further exacerbated sepsis-associated systemic inflammation and organ injury [135].

Since then, we have conducted a series of studies to further explore the role played by H_2_S in sepsis-induced multiple organ dysfunction and to elucidate the underlying mechanism. For example, in mice subjected to CLP-induced sepsis, NF-κB was activated by the elevated H_2_S, leading to an upregulation of the production of proinflammatory cytokines (IL-1β, IL-6 and TNF-α) and chemokines (monocyte chemotactic protein-1, and macrophage inflammatory protein-2), the rolling and adherence of leukocytes, the expressions of various adhesion molecules (intercellular adhesion molecule-1, P-selectin and E-selectin) and eventually exaggerated lung injury and liver injury [136,137]. Thereafter, we found that the extracellular signal-related kinase (ERK) pathway was involved in the activation of NF-κB by H_2_S following sepsis, as the treatment of the ERK kinase inhibitor significantly abolished H_2_S-mediated NF-κB activation and consequently attenuated sepsis-associated systemic inflammation and organ injury [138]. Subsequently, taking the advantage of using the tachykinin precursor 1 gene (the gene that encodes substance P)-deficient mice, as well as using inhibitors of tachykinin receptor 1, the functional receptor of substance P and transient receptor potential vanilloid type 1 (TRPV1), we found that in mice with CLP-induced polymicrobial sepsis, TRPV1-mediated priming of the substance P-tachykinin receptor 1 axis was involved in H_2_S-induced activation of the ERK/NF-κB pathway and further resulted in sepsis-associated alterations, including systemic inflammation and organ injury [139,140,141]. We also found that the involvement of TRPV1-mediated an increase in cyclooxygenase-2 and prostaglandin E metabolite production in H_2_S-induced sepsis-associated alterations in mice [142]. Furthermore, the proinflammatory effect of H_2_S in polymicrobial sepsis was confirmed as the treatment of small interference RNA that targets the CSE gene reduces the accumulation of leukocytes and the levels of proinflammatory mediators in the liver and lungs [143].

More recently, sepsis was induced in genetically deficient CSE-knockout mice and wild-type mice, by which we further shed light on the proinflammatory action of H_2_S in sepsis. The liver sinusoid plays a significant role in maintaining the hepatic function; however, its homeostasis is frequently disrupted in sepsis and endotoxemia. Building on the finding that H_2_S serves as a vasoconstrictor in the liver sinusoid in endotoxemia [144,145], we further explored the effects of H_2_S on liver sinusoid in sepsis. In wild-type mice, sepsis-induced elevated H_2_S caused several alterations in the liver sinusoidal endothelial cells (LSECs), including the defenestration and gaps formation, suggesting that the liver sinusoidal function was impaired by H_2_S in sepsis. However, these alterations were significantly reversed in mice genetically deficient in CSE [146,147]. Furthermore, the underlying mechanisms were investigated. We found that the activation of the ERK1/2-NF-κB p65 pathway and the substance P-tachykinin receptor 1 axis plays a central role in H_2_S-induced liver sinusoidal dysfunction [146,147]. Similarly, aberrant metabolism of H_2_S has been observed in patients with sepsis and animals with LPS-induced endotoxemia [148,149,150].

A potential beneficial effect of a very low dose of H_2_S or slow-release H_2_S donors on sepsis and endotoxemia has also been reported. In mice with CLP-induced sepsis, either prophylactic or therapeutic treatment of H_2_S donors (NaHS or Lawesson’s reagent) promoted the rolling and adhesion of leukocytes and the migration of neutrophils, thereby reducing the bacteremia levels, as well as alleviating hypotension and lung lesions, eventually leading to increased survival. Conversely, the administration of PAG significantly aggravated CLP-induced alterations in mice [151]. It is noteworthy that there were many differences between this study and those discussed above. While this study mainly focused on the role of exogenous H_2_S in sepsis, the research discussed above mainly focused on endogenous H_2_S. In addition, the way in which mice received the treatment of CSE inhibitors and H_2_S donors was also different. This may differ the pharmacological effects of these compounds (subcutaneous injection vs. intraperitoneal injection). Furthermore, the dose of PAG (about 4.5–4.9 g/kg vs. 50 mg/kg) and NaHS (0.56–5.6 mg/kg vs. 10 mg/kg) used in this study and those discussed above was significantly different. These differences may underlie the opposite effects of H_2_S in sepsis reported in these investigations. The protective role of exogenous H_2_S was further confirmed by Ahmad and colleagues as they found that delayed treatment of NaHS was favorable to rats subjected to CLP-induced sepsis [152]. In addition, it is reported that the preservation of mitochondrial function by NaHS treatment resulted in the improvement of diaphragm weakness and the decline of mortality rate in CLP-induced sepsis rats [153]. More recently, two studies have shown that the pretreatment of GYY4137 (25 mg/kg and 50 mg/kg intraperitoneal injection), a novel slow-releasing H_2_S donor, protected against acute lung injury caused by CLP-induced sepsis in mice [154,155]. A similar salutary effect of H_2_S was also reported in urinary-derived sepsis, pseudomonas aeruginosa sepsis and pneumococcal pneumosepsis, together with LPS-induced endotoxemia [21,156,157,158,159].

## 5. Gaseous-Mediator-Based Therapeutic Strategy for Sepsis/Septic Shock

**Figure 3:**
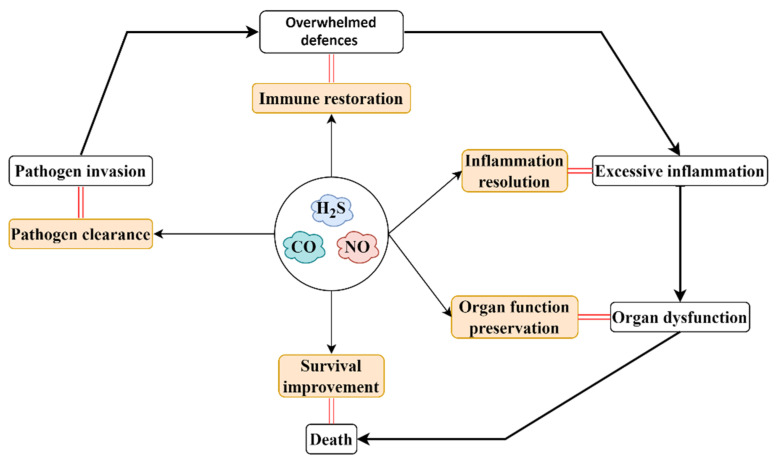
Therapeutic perspective of NO, CO and H_2_S in sepsis. Multiple organ dysfunction resulting from excessive inflammation caused by an aberrant host defense response directed at invasive pathogens and their toxins is one hallmark of sepsis, which can eventually lead to death (as shown in white boxes). To promote the survival of sepsis, several medical countermeasures targeting sepsis pathophysiology, including pathogen clearance, immune restoration, inflammation resolution and organ function preservation, have been developed (as shown in light orange boxes). While the feasibility, safety and efficacy of NO-, CO- and H_2_S-based treatments should be further assessed, the therapeutic promise of gaseous-mediator-based treatments has been observed in many investigations.

The recognition of the involvement of NO, CO and H_2_S in the physiopathology of sepsis/septic shock and endotoxemia has led to the development of therapeutic approaches targeting these gaseous mediators for sepsis (Figure 3). To date, in addition to NO inhalation, many other strategies targeting the regulation of the activity of NOS, the clearance of NO and the bioavailability of the substrate have been widely investigated [105,106,111,160]. Inhaled NO possesses promise in sepsis treatment, since it was reported that systemic oxygenation was improved after NO inhalation [161]; however, inhaled NO (40 ppm) failed to augment microcirculatory perfusion and improve organ function in patients with sepsis [162]. As mentioned by the researchers, one possible reason for the failure of inhaled NO in sepsis is that the macrocirculatory hemodynamics of these patients had been optimized prior to the treatment of inhaled NO. Considering the deleterious effects of overproduction of endogenous NO in sepsis, several NOS inhibitors, including selective and nonselective, have been developed, and the therapeutic efficacy and safety of these compounds have been widely investigated. Unfortunately, these studies have produced mixed results; thus, whether to treat sepsis by NOS inhibitors is still a matter of debate [111,163]. While a phase II trial showed that L-NMMA, a nonselective NOS inhibitor, improved systemic vascular response in patients with septic shock, a phase III trial conducted subsequently was terminated. as L-NMMA increased mortality in patients with septic shock [164,165]. The larger phase III investigation was thought to reveal an adverse outcome of L-NMMA treatment that was not detected by the smaller phase II study [165]. In the reactions leading to the NOS-catalyzed production of NO, BH4 does not only act as a crucial cofactor, but it also serves as an endogenous regulator of NOS activity. Overproduction of BH4 and an attendant increase in conversion of BH4 to BH2 result in a high level of BH2 in sepsis. BH2 can bind to NOS and consequently suppress the activity of NOS, leading to a decrease in NO biosynthesis [166]. Promisingly, the administration of the BH4 analog attenuated hemodynamic instability, organ dysfunction and declined mortality in animals with sepsis or endotoxemia [111,163]. Similarly, several novel NO-based therapeutic strategies targeting the enhancement of NO clearance or the improvement of NO bioavailability have been developed for sepsis [111].

The recognition of the beneficial effects of CO in sepsis in animal models has prompted the development of CO-based therapy for sepsis. The administration of low-dose inhaled CO showed a protective effect in sepsis, as it has been shown to rescue mice from severe sepsis induced by *Staphylococcus aureus* infection [125]. More recently, the feasibility and safety of low-dose inhaled CO administration in patients with sepsis-induced acute respiratory distress syndrome were established in a phase 1 trial [167]. In addition, this study also demonstrated that the administration of low-dose inhaled CO significantly reduced the concentrations of mitochondrial DNA in the plasma, indicating the potential effect of inhaled CO treatment in preserving mitochondrial function in sepsis [167]. Taking advantage of the knowledge that exogenous CO releases from CORMs, including CORM-1, CORM-2 and CORM-3, have potent protective roles in sepsis and endotoxemia, increasing attention has been paid to treating animals with sepsis using these novel compounds [120,121].

Accumulating evidence has revealed the complicated actions played by H_2_S in sepsis, leading to the increasing attention being paid to develop therapeutic approaches targeting H_2_S for sepsis. Although several preclinical animal studies have shown the protective role of using PAG in sepsis [136,141], many other investigations have also indicated that the administration of inhaled H_2_S or H_2_S donors, either fast-releasing donors or slow-releasing donors, such as NaHS and GYY4137, is beneficial to sepsis and endotoxemia, resulting in uncertainties in the possible role of H_2_S-based treatment for sepsis [20,152,153,154,155]. Given the promise of the countermeasures targeting gaseous mediators in sepsis therapy, the establishment of an optimal therapeutic protocol, including the dose and delivery, for gaseous-mediator-based therapy for sepsis will be meaningful and another step forward.

## 6. Conclusions

In sepsis, a dysregulated host defense response directed at invasive pathogens and their toxins can cause excessive systemic inflammation, consequently leading to multiple organ dysfunction and death. Accumulating evidence, from our laboratory and others, has revealed the important roles of NO, CO and H_2_S as novel mediators in regulating the onset, development, progression and outcome of sepsis. Furthermore, the understanding of the significant roles of NO, CO and H_2_S in the pathophysiology of sepsis, including their effects on host immune response, pathogen elimination, systemic inflammation and organ dysfunction, has led to the development of several novel therapeutic strategies targeting NO, CO and H_2_S for sepsis, such as inhaled NO, CO and H_2_S, the inhibitors of NOS, HO and CSE and the slow-releasing donors of NO, CO and H_2_S. Although more research is needed to evaluate the feasibility, safety and efficacy of these gaseous-mediator-based treatments, early results have shown the promise of these novel therapeutic strategies. Thus, it is important to put more effort and resources in order to investigate the therapeutic prospects of NO, CO, and H_2_S in sepsis.

## Figures and Tables

**Table 1:** Characteristics of currently recognized gaseous mediators.

	Nitric Oxide	Carbon Monoxide	Hydrogen Sulfide
Formula and molecular weight	NO (30.01 g/mol)	CO (28.01 g/mol)	H_2_S (34.08 g/mol)
Biological half-life	Seconds	Several minutes	Seconds–minutes
Chemical reactivity	Very high	Moderate	Very high
Properties of free radicals	Yes	No	Yes
Endogenous production enzymes	nNOS; iNOS; eNOS; mtNOS ^a^	HO-1; HO-2; HO-3 ^a^	CBS; CSE; 3-MST/CAT; 3-MST/DAO ^b^
Main substrates for biosynthesis	L-arginine	Heme	L-cysteine; 3-mercaptopyruvate; D-cysteine ^b^
Clearance sources	Oxidization	Being exhaled from the airway; Binding to heme proteins; Oxidization ^c^	Oxidization (mitochondrion); Methylation (cytoplasm); Being excreted from urine
End products	Nitrite and nitrate	Carboxyhemoglobin; Carbon dioxide ^c^	Thiosulfate and sulfate; methanethiol
Second messenger	sGC	sGC	NA
Involvement in sepsis	Yes (Mainly detrimental)	Yes (Mainly beneficial)	Yes (Mainly detrimental)

Abbreviations: neuronal nitric oxide synthase (nNOS); inducible nitric oxide synthase (iNOS); endothelial nitric oxide synthase (eNOS); mitochondrial nitric oxide synthase (mtNOS); heme oxygenase-1 (HO-1); heme oxygenase-2 (HO-2); heme oxygenase-3 (HO-3); cystathionine β-synthase (CBS); cystathionine γ-lyase (CSE); 3-mercaptopyruvate sulfurtransferase (3-MST); cysteine aminotransferase (CAT); D-amino acid oxidase (DAO); soluble guanylyl cyclase (sGC); Not applicable (NA). ^a^ Controversial; ^b^ newly discovered; ^c^ has not been observed under physiological conditions.

## Data Availability

Not applicable.
